# The Clinical and Radiological Outcomes and Complications of Bilboquet Implant for Proximal Humerus Fractures: A Systematic Review

**DOI:** 10.3390/jcm13237398

**Published:** 2024-12-04

**Authors:** Ramy Samargandi, Jawad Albashri, Ahmed Albashri, Faris Alzahrani, Abdulrahman Hassan, Julien Berhouet

**Affiliations:** 1 Department of Orthopedic Surgery, College of Medicine, University of Jeddah, Jeddah 23218, Saudi Arabia; 2 Service de Chirurgie Orthopédique et Traumatologique, Centre Hospitalier Régional Universitaire (CHRU) de Tours, 1C Avenue de la République, 37170 Chambray-les-Tours, France; 3 College of Medicine, University of Jeddah, Jeddah 23218, Saudi Arabia

**Keywords:** bilboquet implant, proximal humerus fractures, clinical outcomes, radiological outcomes, complications

## Abstract

**Background/Objectives:** There is no consensus in the literature regarding the optimal treatment for complex proximal humerus fractures (PHFs). The aim of this study is to evaluate the clinical, functional, and radiological outcomes, and complications, associated with the Bilboquet implant in the treatment of PHFs. **Methods:** The search was conducted from the first description of the Bilboquet device in 1994 to June 2024, across PubMed, Web of Science, and Google Scholar, using specific keywords such as (“Bilboquet” OR “Bilboquet prosthesis” OR “Bilboquet device”) AND (“proximal humerus fracture” OR “shoulder fracture”), along with Boolean operators. The inclusion criteria comprised studies published in English or French that focused on the use of the Bilboquet implant for PHFs. Eligible study designs included case series, cohort studies, randomized controlled trials (RCTs), and non-RCTs evaluating clinical, functional, and radiological outcomes, and complications. Studies that do not contain relevant results to this systematic review, pediatric populations, or the use of alternative implants were excluded. **Results:** A total of eight studies (235 patients) published between 1996 and 2021 were included. The mean age was 68.6 years (56 to 76.8) in all the studies. The majority of patients, 76.2%, were females, with male patients accounting for only 23.8%. A total of 10 (4.3%) patients had 2-part fractures, 40% of patients had 3-part fractures, and 55.7% of patients had 4-part fractures. The mean follow-up was 36.4 months (25.8–88.7), with a mean constant score of 69.7 (62–78.6). Complications included non-union in 2.65% of cases, avascular necrosis in 19.7%, revision surgery in 5.1%, and protrusion of the staple in 4.3%. **Conclusions:** Despite limited knowledge of the Bilboquet implant, it shows promise in managing complex PHFs in both young and older adults, with favorable clinical and radiological outcomes. It offers advantages over traditional fixation methods and allows easy conversion to arthroplasty if osteonecrosis occurs. However, the long-term outcomes require further study. While early results are promising, larger randomized studies are needed to confirm its broader clinical utility.

## 1. Introduction

Proximal humerus fractures (PHFs) are the third most common non-vertebral fracture and the second most common fracture in the upper extremity, comprising 4 to 6% of all fractures in the elderly population [1]. The lifetime risk of fracture for women aged 50 is approximately 13%, with nearly half of these patients having experienced prior fracture [2]. Between 1970 and 2002, the incidence of PHFs tripled [3]. For women over the age of 80, the incidence reached as high as 520 per 100,000 per year, with displaced 3- and 4-part fractures accounting for 14% of the total prevalence [4,5]. This increase in PHF incidence is becoming more common worldwide due to aging populations [6]. Consequently, due to their high prevalence and the projected rise in incidence, these fractures pose a substantial burden on healthcare systems [7].

While most fractures are minimally displaced and could be managed conservatively [8], surgical treatment is typically considered for unstable displaced 3- and 4-part fractures [9]. Many treatment approaches have been described, including closed reduction, open reduction and internal fixation (ORIF), percutaneous pinning, and shoulder arthroplasty either by hemiarthroplasty (HA) or reverse shoulder arthroplasty (RSA) [10,11]. Until now, there seems to be no consensus in the literature regarding the optimal treatment for complex PHFs. The two most popular techniques for treating complicated PHFs are ORIF and shoulder arthroplasty.

Locking plates and intramedullary nailing (IMN) are the most prevalent ORIF modalities. Outcome studies using locking plates continue to yield mixed outcomes, with complication rates as high as 20–30% and a reoperation rate of 10% [12,13]. However, there is a substantial failure risk when applied alone in cases of posteromedial comminution, metadiaphyseal fracture extension, and non-unions [14]. The IMN provides less soft tissue dissection, causes less injury to the periosteum and blood vessels, and can provide minimally invasive fixation [15]. However, according to several studies, there were no differences between treatments in constant score or postoperative complications [16,17]. Furthermore, studies have shown poorer outcomes in patients treated with an IMN for 3- or 4-part fractures [18,19]. Also, wide variations exist in the outcomes of HA. The results were less than satisfactory in some studies [20,21], whereas some authors reported an acceptable outcome [22,23]. The usage of the RSA has increased, particularly in cases of 3- and 4-part fractures, according to a study by Kelly et al. [24], in revision and reoperation. The range of motion (RoM) and satisfaction scores are higher than in other surgical approaches. Despite advancements in these techniques, challenges in achieving optimal fixation and minimizing complications remain.

**Figure 1:**
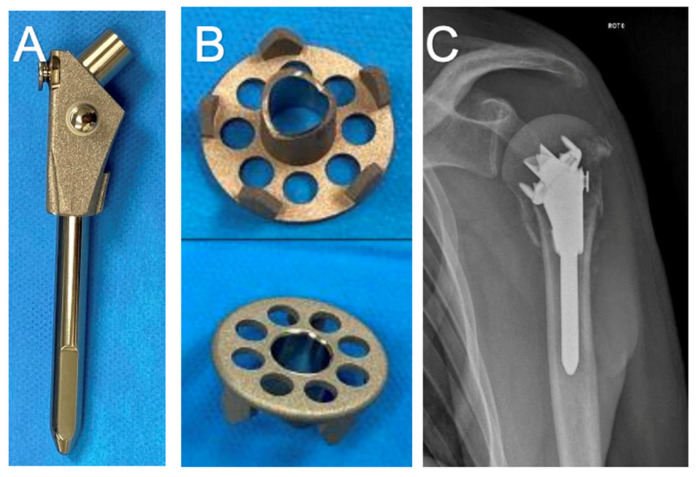
The Bilboquet implant: (**A**) JUST cementless stem (Evolutis, Briennon, France), (**B**) head staple component, (**C**) X-ray demonstrating Bilboquet implant.

In this context, the Bilboquet system, described by L. Doursounian in 1996, offers a novel approach to PHF fixation [25]. This technique is primarily indicated for complex PHFs, particularly three-part and four-part fractures. This Bilboquet system uses a staple with a female Morse cone fixed in the humeral head and an intramedullary cemented or cementless stem with a male Morse cone that fits the cephalic staple [26], as demonstrated in Figure 1. The procedure involves exposing the fracture through either a deltoid-splitting or deltopectoral approach, followed by careful elevation of the humeral head. The staple is impacted into the cancellous bone of the humeral head, and the stem is inserted into the humeral shaft. The Morse taper of the stem is then engaged with the staple’s female taper, as detailed by L. Doursounian [27,28]. This system’s modular design preserves humeral morphology, reduces varus tilt, and facilitates easier conversion to arthroplasty, addressing the key limitations of traditional fixation methods. These attributes make the Bilboquet system a promising alternative for managing complex PHFs, especially in cases requiring stable fixation and potential revisions. Many studies have shown this implant’s efficacy for treating PHFs with good clinical and functional outcomes [26,27,28,29,30].

Despite these positive results, the Bilboquet device is not widely commercialized, utilized, or recognized globally. The existing literature includes a variety of case series and small cohort studies, most of which have been published in France [26,28,30,31]. Moreover, its limited global adoption could be attributed to factors such as a lack of awareness, limited access to the device outside of certain regions, and the availability of more commonly used alternatives like IMN or locking plates, which are already well established in the surgical community. Additionally, the relatively small body of evidence supporting this technique compared to other methods may also contribute to its limited use worldwide, but a thorough synthesis of the medical literature is needed to draw more definitive conclusions.

This systematic review aims to evaluate the effectiveness; clinical, functional, and radiological outcomes; and complications associated with the Bilboquet implant in treating PHFs. By reviewing and analyzing data from various studies, we aim to provide a clearer understanding of the benefits and limitations of this implant. We hypothesize that the Bilboquet implant is an effective treatment for managing PHFs in both young and older adults with significant bone loss.

## 2. Materials and Methods

### 2.1. Literature Search

This systematic review was conducted with strict adherence to the Preferred Reporting Items for Systematic Reviews and Meta-Analyses (PRISMA) guidelines [32]. The literature search covered the period from the first description of the Bilboquet device in 1994 to June 2024, utilizing PubMed, Web of Science, and Google Scholar to ensure a comprehensive and robust search strategy. The databases were accessed through Rayyan, and specific keywords such as (“Bilboquet” OR “Bilboquet prosthesis” OR “Bilboquet device” OR “prothèse Bilboquet”) AND (“proximal humerus fracture” OR “shoulder fracture” OR “fracture de l’humérus proximal” OR “fracture de l’épaule”) were used. Boolean operators “OR” and “AND” were employed to refine the search and obtain precise, relevant results for the study.

### 2.2. Study Selection

The target population was patients with PHFs treated with the Bilboquet implant. Outcomes assessed included clinical, functional, and radiological results, and complications. The inclusion and exclusion criteria were as follows:

Inclusion Criteria

- Studies evaluating the use of the Bilboquet device for the treatment of PHFs.
- Articles published in English or French up to June 2024.
- Study designs including case series, prospective and retrospective cohort studies, randomized controlled trials (RCTs), and non-RCTs.
- Outcomes assessed included clinical, functional, and radiological results, and complications.

Exclusion Criteria

- Studies involving pediatric populations or patients under 18 years of age.
- Articles reporting on fractures treated with devices other than the Bilboquet implant.
- Case reports, consensus meetings, conference abstracts, and studies lacking relevant results to this systematic review.
- Non-English or non-French articles.

### 2.3. Data Extraction

Full-text articles were retrieved and screened, and two investigators retrieved data from each report. General information was collected from the article including the title, author, study design, year of publication, country, name of the journal that published, number of patients, sex, age, follow-up time, side of fracture, Neer classification (number of fracture parts), implant company, type of stem used (cemented or cementless), functional scores when available including RoM, constant score, Disabilities of the Arm, Shoulder, and Hand (DASH) Score, American Shoulder and Elbow Surgeons (ASES) Score, and the rating category system (Duparc and Huten) [33], which categorizes outcomes into four levels: excellent, good, fair, and poor. These categories are based on pain levels and active forward elevation (AFE). An “excellent” outcome indicates slight or no pain with an AFE of 120° or more. A “good” outcome denotes slight or moderate pain that does not interfere with function and an AFE between 90° and 120°. A “fair” outcome is characterized by moderate pain that interferes with function, with an AFE between 60° and 90°. Lastly, a “poor” outcome indicates disabling pain, with an AFE below 60°. Complications reviewed included protrusion of staple, infection rates, avascular necrosis (AVN), non-union, malunion, tilt rates, revision rates, and type of revision. Regarding the discrepancy between reviewers, there were no disagreements between the reviewers.

### 2.4. Risk of Bias Assessment

Since no RCTs were found in the literature search and most studies were case series, all the studies were carefully evaluated for potential bias and the quality of evidence. The case series were evaluated using a tool suggested by Murad et al. [34], which adapts the Newcastle–Ottawa Scale and causality criteria specifically for case series. The tool consists of 8 questions representing 4 domains: Selection, Ascertainment, Causality, and Reporting. The risk of bias was reported for each of these 4 domains. Also, cohort, case–control, and comparative observational studies were assessed using the Methodological Index for Non-Randomized Studies (MINORS) [35], a validated tool designed to evaluate the methodological quality of non-randomized studies. The tool comprises 12 items for non-comparative studies and 8 items for comparative studies, with each item scored from 0 to 2. The maximum score is 16 for non-comparative studies and 24 for comparative studies. Two authors independently evaluated the quality of the included studies, resolving any disagreements through discussion or by consulting a third author.

### 2.5. Statistical Analysis

Descriptive statistics were used to summarize patient demographics, fracture types, and outcomes across the included studies. Mean values and ranges were calculated for variables such as age, follow-up duration, functional scores, and RoM. Complication rates, including avascular necrosis, non-union, malunion, revision surgeries, varus tilt, and staple protrusion, were expressed as percentages of the total sample.

## 3. Results

**Figure 2:**
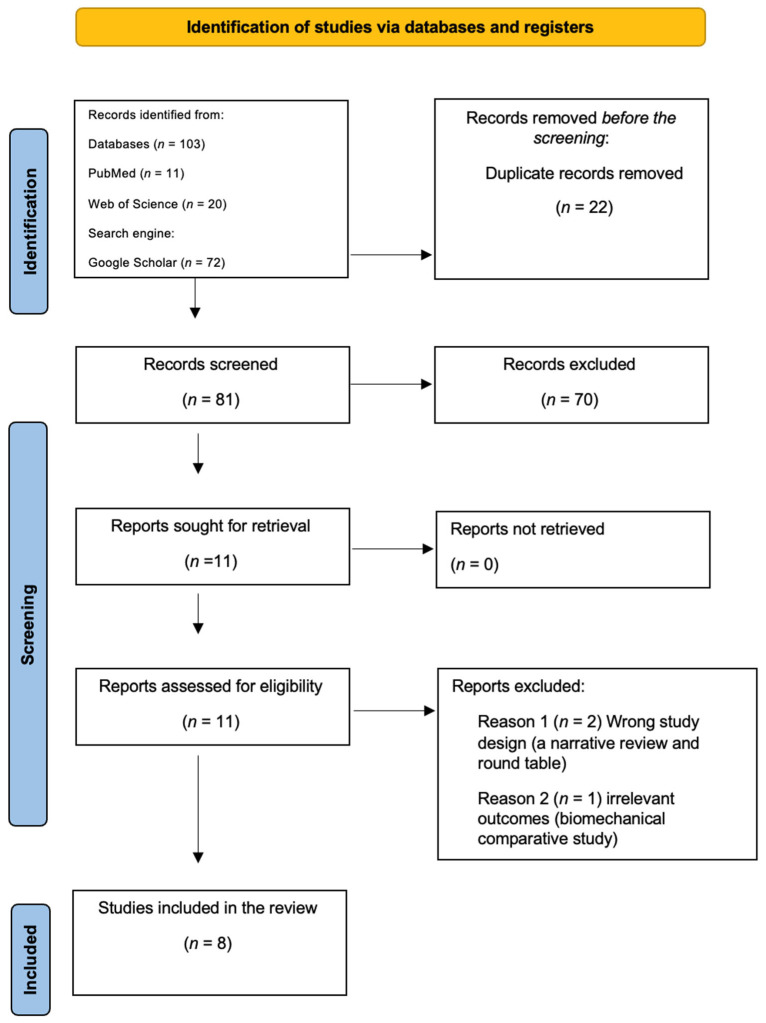
PRISMA flow diagram.

A flow diagram of the study selection process is presented in Figure 2, and a total of 103 studies were identified through database searches and search engines such as Web of Science, Google Scholar, and PubMed. The duplicated records that were removed were 22 articles before the screening. A total of 81 articles were subsequently screened by the title and the abstract. Eleven studies were reviewed in full text to assess the eligibility criteria, and three studies were excluded, as described in the PRISMA figure. A total of eight articles were included in this systematic review [25,26,27,28,29,36,37,38].

### 3.1. Study Characteristics

Table 1 shows the demographic characteristics of participants, the type of implant used, and the Neer classification. A total of 235 patients were included in the eight studies [25,26,27,28,29,36,37,38]. The majority of the studies were case series (*n* = 7) [25,26,27,29,36,37,38], whereas six studies were retrospective studies [25,26,27,28,37,38] and two studies were prospective studies [29,36]. Apart from one study that was conducted in India [29], all other studies were conducted in France [25,26,27,28,36,37,38]. The mean age was 68.6 years, which ranged from 56 to 76.8 years in all the studies, with five out of eight studies reporting a mean age above 70 years. The majority 76.2% of patients were females (*n* = 179), with male patients accounting for only 23.8% (*n* = 56). Regarding the type of stem, four (50%) studies used a cement-type stem [25,26,36,37] whereas the other 50% used a cementless stem [27,28,29,38], with a total of 126 patients (53.6%) having cemented stems and 109 patients (46.3%) having cementless stems. Right-side shoulder involvement was seen in 88 (37.5%) patients, whereas 64 (27.2%) patients had left-side shoulder involvement. A total of 10 (4.3%) patients had two-part fractures, 94 (40%) patients had three-part fractures, and 131 (55.7%) patients had four-part fractures, according to Neer classification.

### 3.2. Quality Assessment of the Included Studies

Regarding the studies (*n* = 7) [25,26,27,29,36,37,38] that were assessed by Murad et al.’s tool [34], there was no risk of bias regarding selection (Q1) in any of the included studies. Similarly, no risk of bias was reported regarding the ascertainment parameters in any of the included studies in this systematic review (Q2 and Q3). Regarding causality, no study reported other alternative causes that may explain the observations that were ruled out (Q4). Similarly, no study reported a challenge/rechallenge phenomenon (Q5). No study reported a dose–response effect (Q6). There was no risk of bias regarding reporting outcomes (Q8). Only one study [28] was assessed with the MINORS tool [35]. The total score was 18. Two items in the MINORS assessment pertaining to the prospective collection of data and prospective calculation of the study size were not reported. Similarly, two items relating to the unbiased assessment of the study endpoint and baseline equivalence of groups were reported but inadequate. The remaining items related to a clearly stated aim, the inclusion of consecutive patients, endpoints appropriate to the aim of the study, a follow-up period appropriate to the aim of the study, a loss to follow-up of less than 5%, an adequate control group, contemporary groups, and adequate statistical analysis were adequality reported (Table 2 and Table 3).

### 3.3. Clinical and Radiological Outcomes

The mean follow-up duration was 36.4 months (Range: 25.8–88.7) in the included studies whereas the mean constant score was 69.7 (Range: 62–78.6). Moreover, studies conducted by Doursounian et al. [25,26,37] employed a rating system described by Duparc and Huten [33]. Across the three studies, which included a total of 104 cases, 35 patients (33.8%) had excellent outcomes, 34 (32.8%) had good outcomes, 25 (24%) had fair outcomes, and 10 (9.6%) had poor outcomes. Regarding the RoM, the AFE ranged from 100° to 137.3°, with an average of 108.25° in the included studies. The external motion ranged from 22° to 38.67° whereas the internal motion ranged from 2.3 to 6.56 points (Table 4).

Regarding complications, osteonecrosis was observed in 46 (19.65%) patients whereas revision procedure was performed in 12 (5.11%) patients. HA was reported as a revision procedure in all of the patients except for one case that underwent RSA. Protrusion of the staple was in 10 (4.32%) patients. Moreover, the non-union rate was observed in six (2.65%) of the patients, while the infection rate was 0.43%, as it was reported in only one case [28]. Malunion was reported in four (1.70%) cases in only one study [36]; meanwhile, varus tilt was seen in ten (4.32%) cases from five studies (Table 5).

## 4. Discussion

Comminuted PHFs are often associated with a loss of bone stock in the tuberosities and metaphysis, making adequate reduction and stable fixation challenging. Internal fixation with plate osteosynthesis techniques is not always suitable in such cases, as it is associated with high complication rates, particularly in the presence of osteoporosis [39]. The correct morphological pattern of the proximal humerus can be achieved through the restoration of the distance between the shaft and head. In such cases, even intramedullary devices do not provide the adequate support needed for the humerus head. Therefore, varus tilt or the penetration of hardware in the head is usually seen in such cases. Due to these difficulties, the surgeons often resort to arthroplasty. In the Bilboquet technique, the spacing between the shaft and the head is preserved due to the central and, particularly, the peripheral support on the humeral head, which prevent any varus displacement. After securing the tuberosities onto the hardware, they come in contact with bone [37]. Achieving an anatomical reduction in the humeral head to the diaphysis promotes proper alignment of the tuberosities, which tend to position themselves accurately during fixation. This consistent union is one of the key benefits of the Bilboquet system [36].

Another advantage of the Bilboquet system over HA is that tuberosity fixation can be performed on a bone-to-bone interface, optimizing the chances of union, which is a critical factor in functional outcomes [40,41]. The high success rates of tuberosity union, with a low incidence of non-union in the Bilboquet system, underscore the effectiveness of this implant. In this systematic review, the incidence of non-union was observed to be 2.55%, which is still acceptable in comparison with HA.

The Bilboquet implant demonstrates a lower incidence of varus tilt compared to traditional internal fixation methods and was observed in 10 patients (4.2%). Varus tilt can significantly impair shoulder function and lead to poor clinical outcomes [42]. The role of medial support in the maintenance of fracture reduction is essential when considering the proximal humeral plate as a method of fixation. The absence of medial support can lead to a loss of reduction and carry a risk of screw penetration and subsequent AVN, especially in cases with humeral plate fixation [43,44]. This can be prevented by providing medial support using a fibular graft, which increases the complexity of the surgery and raises the morbidity risk for the patients [45,46]. However, it may be more effective to use a nail rather than a plate or Bilboquet implant, which functions biomechanically like a nail to stabilize the medial deficiency and avoid collapse. This approach may offer a less complex and lower-risk alternative for maintaining medial support and achieving better outcomes.

When AVN occurs, converting to HA using the Bilboquet implant is significantly easier. The design of the implant facilitates the removal and replacement process with an HA component, maintaining the integrity of the humeral head even if AVN sets in. The stem’s Morse taper cone can be fitted into the staple for internal fixation or into a humeral head prosthesis if HA is necessary. However, the conversion of internal fixation with the Bilboquet system to HA is not as common as initially anticipated [37]. Despite this fact, the procedure is still much easier compared to conventional internal fixation [37]. The incidence of AVN associated with the Bilboquet implant is generally low, and in cases where AVN occurs, it is often tolerable due to the healing of the tuberosities. In the present systematic review, necrosis was reported in 46 cases and only 12 cases needed revision.

One possible advantage of the Bilboquet implant is its ability to reduce radiation exposure for the patients and the surgeon compared to traditional internal fixation methods [26], which often require multiple radiographic assessments to ensure proper alignment and fixation [47,48,49]. Unlike other techniques, the Bilboquet system minimizes the need for precise screw and plate placement, thereby lowering the risk of intra-articular screw penetration [50], which occurs in 11% to 30% of cases due to inaccurate measurements, inadequate fluoroscopic control, or complications such as varus collapse or humeral head necrosis [51,52]. Intra-articular screw penetration can be difficult to confirm on X-rays. Some authors suggest using postoperative CT scans to evaluate intra-articular screw penetration in PHFs, especially when radiographic assessment is uncertain. However, this approach may further increase radiation exposure [53]. The Bilboquet implant simplifies the fixation process, reducing both the operative time and procedural complexity [26].

Another advantage of the Bilboquet implant in complex PHFs is its potential to avoid prosthetic replacement by preserving the humeral head, which may positively influence psychological outcomes. In a prospective study comparing psychological outcomes between osteosynthesis and RSA for the treatment of PHFs, osteosynthesis demonstrated better General Anxiety Disorder-7 (GAD-7) and Caregiver Strain Scale (CSS) scores at 12 months postoperatively, emphasizing the psychological benefits of osteosynthesis over arthroplasty [54].

Regarding the use of the Bilboquet implant in elderly patients, it remains a debatable topic. For example, a review by Lascar et al. [31] stated that in osteoporotic patients over the age of 80 with four-part fractures, the use of the Bilboquet implant is an error of indication, as it carries a high risk of AVN, which may require revision surgery. In such cases, RSA is considered the best option for this age group, providing an acceptable functional outcome. Additionally, Le Dû and Favard [30] concluded that the Bilboquet system is a method of choice for the fixation of complex PHFs, except in cases where fractures pose a high risk of AVN, particularly in older patients. They demonstrated that patients who developed AVN had worse constant scores and suggested that early revision with HA should be considered before staple protrusion occurs. In contrast, Doursounian et al. [37] reported positive outcomes using the Bilboquet device for PHFs in elderly patients. Their study concluded that patients aged 66 to 95 years (mean age = 76.8) are suitable candidates for reduction and fixation using the Bilboquet technique. We believe that RSA is the best option for elderly patients over 80 compared to the Bilboquet. This is despite our review showing that most patients who developed AVN did not require further revision surgery, and revisions are relatively easy without the need to change the stem, as it is adaptable for all types of shoulder prostheses. In our opinion, the Bilboquet technique is most appropriate for younger elderly patients aged 60 to 79 years with three-part or four-part PHFs and should not be used in patients over 80 years old with three-part or four-part PHFs, except in cases where the prognosis for vascular perfusion of the humeral head is favorable. Furthermore, in younger patients with complex PHFs, when the decision between prosthetic surgery and internal fixation is debated, the Bilboquet remains a highly appealing alternative treatment option, as it offers higher tuberosity union rates compared to immediate hemiarthroplasty and potentially easier management of complications [31].

Initially, the Bilboquet implant faced challenges related to the size of the staples, limiting its applicability across different patient anatomies [27]. However, advancements in the design have led to the availability of multiple staple sizes [27]. This improvement has broadened the scope of the Bilboquet implant in clinical practice. The development of cementless stem options has further expanded the utility of the Bilboquet implant. Cementless stems offer the advantage of biological fixation, potentially leading to better long-term outcomes by promoting osseointegration and reducing the risk of implant loosening [27].

Although the Bilboquet implant offers several advantages, its successful implementation requires a certain level of surgical expertise, particularly for staple fixation and achieving proper retroversion alignment. Errors in these steps may lead to complications, such as varus tilt or poor functional outcomes. As such, adequate training and experience are essential for the effective use of this implant [37]. Additionally, one of the limitations of the Bilboquet implant is that once it is inserted, it cannot be easily removed without sacrificing the humeral head. This irreversible nature necessitates careful patient selection and preoperative planning [37]. Recent advancements in orthopedic surgery, such as 3D CT scan reconstruction and 3D printing, can address these challenges by enabling precise planning, enhancing the understanding of fracture fragments, and aiding in proper surgical decision-making, thereby potentially optimizing outcomes and reducing complications [55,56].

This systematic review has several limitations that should be considered when interpreting its findings. The primary limitation is the small number of included studies, most of which were case series or retrospective studies, with the largest involving only 61 patients [37]. Additionally, all studies, except one, were conducted in France, with six out of the eight studies authored by the same research group, limiting the generalizability of the findings. The lack of large-scale, randomized studies comparing the Bilboquet implant with other fixation methods or arthroplasties is another limitation. The simplifications used in this systematic review include the reliance on heterogeneous outcome measures across included studies. These simplifications may have introduced selection bias. While current studies support the efficacy of the Bilboquet implant, long-term follow-up data are lacking. For instance, only one study by Bismuth et al. [28] compared it with a locking plate, demonstrating better functional results at two years. To address these limitations, future research should focus on larger, multicenter RCTs to compare the Bilboquet implant with other fixation techniques and arthroplasties, focusing on both clinical and radiological outcomes. Additionally, further studies should explore the standardization of surgical techniques to reduce complications and evaluate long-term outcomes to confirm the durability of the implant. The development of training protocols for surgeons to ensure proper use of the device could also help mitigate the reliance on surgical expertise. Finally, including broader populations and diverse geographic settings in future studies will enhance the applicability of findings. Despite these limitations, this is the first systematic review to comprehensively evaluate the postoperative outcomes of the Bilboquet implant, providing valuable insights into its role in managing complex PHFs.

## 5. Conclusions

Despite the lack of knowledge of the Bilboquet implant worldwide, the Bilboquet implant shows promise in managing PHFs in both young and older adults with significant bone loss. Its design minimizes varus tilt, reduces radiation exposure, and facilitates tuberosity union, offering advantages over traditional internal fixation methods. Additionally, it provides the advantage of easy conversion to HA in case of osteonecrosis, making it a potential fixation device in the management of complex PHF. However, its effectiveness remains dependent on surgical expertise, and the long-term outcomes require further investigation. While early results are favorable, larger RCTs are needed to confirm these findings and establish their broader clinical utility.

## Figures and Tables

**Table 1:** Summary of the included studies.

Study No.	Author, Year	Country	Study Design	Patients (*n*)	Age (Years)	Male: Female (*n*)	Type of Implant	Type of Stem Used	Neer Classification (How Many Parts Fractured)		
									Two Parts	Three Parts	Four Parts
1	Doursounian et al., 2011 [36]	France	Prospective study	22	70 (45–84)	4:18	Stryker Howmedica, France	Cement	0	7	15
2	Doursounian et al., 2005 [37]	France	Case series	61	76.8 (66–95)	6:55	Stryker Howmedica, France	Cement	10	27	24
3	Doursounian et al., 2000 [26]	France	Case series	26	70.4 (27–93)	3:23	Stryker Implants, Cestas, France	Cement	0	16	10
4	Doursounian et al., 2016 [27]	France	Case series	25	67 (44–88)	11:14	JUST UNIC cementless stem (Evolutis, Briennon, France)	Cementless	0	10	15
5	Bismuth et al., 2021 [28]	France	Retrospective study	22	66.5 (40–90)	10:12	JUST UNIC cementless stem (Evolutis, Briennon, France)	Cementless	0	9	13
6	Doursounian et al., 1996 [25]	France	Retrospective case series	17	70 (27–93)	4:13	Stryker France, Cestas, France	Cement	0	10	7
7	Desai et al., 2021 [29]	India	Prospective study	30	56 (43–66)	10:20	(JUST UNIC, Evolutis, France)	Cementless	0	0	30
8	Doursounian et al., 2020 [38]	France	Case series	32	72 (50–84)	8:24	JUST UNIC^®^ (Evolutis, France)	Cementless	0	15	17

**Table 2:** The methodological quality of case reports and case series tool (*n* = 7).

Domain For Evaluating the Methodological Quality of Case Reports and Case Series								
	Selection	Ascertainment		Causality		Reporting		
	Leading Explanatory Questions							
Reference	Q1	Q2	Q3	Q4	Q5	Q6	Q7	Q8
Doursounian, 2011 [36]	Yes	Yes	Yes	Partially	No	No	Yes	Yes
Doursounian, 2005 [37]	Yes	Yes	Yes	No	No	No	Yes	Yes
Doursounian, 2000 [26]	Yes	Yes	Yes	No	No	No	Yes	Yes
L. Doursounian, 2016 [27]	Yes	Yes	Yes	No	No	No	Yes	Yes
Desai S., 2020 [29]	Yes	Yes	Yes	No	No	No	Yes	Yes
L. Doursounian, 2020 [38]	Yes	Yes	Yes	No	No	No	Yes	Yes
L. Doursounian, 1996 [25]	Yes	Yes	Yes	No	No	No	Yes	Yes
**Selection:** [question 1]. Does the patient(s) represent(s) the whole experience of the investigator (center) or is the selection method unclear to the extent that other patients with similar presentations may not have been reported? **Ascertainment:** [question 2]. Was the exposure adequately ascertained? [question 3]. Was the outcome adequately ascertained? **Causality:** [question 4]. Were other alternative causes that may explain the observation ruled out? [question 5]. Was there a challenge/rechallenge phenomenon? [question 6]. Was there a dose–response effect? [question 7]. Was follow-up long enough for outcomes to occur? **Reporting:** [question 8] Is the case(s) described with sufficient details to allow other investigators to replicate the research or to allow practitioners to make inferences related to their own practice?								

**Table 3:** MINORS assessment tool for non-randomized comparative studies (*n* = 1).

Item	Bismuth, 2021 [28]
A clearly stated aim	2
Inclusion of consecutive patients	2
Prospective collection of data	0
Endpoints appropriate to the aim of the study	2
Unbiased assessment of the study endpoint	1
Follow-up period appropriate to the aim of the study	2
Loss to follow-up less than 5%	2
Prospective calculation of the study size	0
An adequate control group	2
Contemporary groups	2
Baseline equivalence of groups	1
Adequate statistical analyses	2
Total score	18

**Table 4:** Clinical and functional outcomes.

Author, Year	Mean FU (Months)	Assessment Score Used	Mean of Evaluated Score	Mean Range of Motion			
				AFE	Abd	ER	IR *
Doursounian et al., 2011 [36]	34	Constant	66	108	85	28	4.9
Doursounian et al., 2005 [37]	30.7	Global rating score	22 Excellent, 19 Good, 15 Fair, 5 Poor.	111	-	26	5.27
Doursounian et al., 2000 [26]	25.9	Global rating score	9 Excellent, 9 Good, 6 Fair, 2 Poor.	112.3	-	28.46	6.3
Doursounian et al., 2016 [27]	26.8	Constant	62	113.8	-	28.8	6.56
Bismuth et al., 2021 [28]	30	Constant DASH	78.6 37.4	137.3	117.5	36.4	2.3
Doursounian et al., 1996 [25]	29	Global rating score	4 Excellent, 6 Good, 4 Fair, 3 Poor.	100		22	6.3
Desai et al., 2021 [29]	25.83	Constant ASES	73.9 75.2	114	96.17	38.67	4.93
Doursounian et al., 2020 [38]	88.7	Constant	68	-	-	-	-

DASH: Disabilities of the Arm, Shoulder and Hand. ASES: American Shoulder and Elbow Surgeons Standard Shoulder Assessment score. AFE: Active Anterior Elevation, ER: External Rotation, IR: Internal Rotation. * Thigh = 0/Buttock = 2/Sacrum, L4, L5 = 4/L1, L2, L3 = 6/T8–T12 = 8/T2–T7 = 1.

**Table 5:** Complications and authors’ conclusion.

Author, Year	AVN (*n*)	Non-Union (*n*)	Varus Tilt (*n*)	Protrusion of Staple (*n*)	Malunion (*n*)	Revision (*n*)	Conclusion
Doursounian et al., 2011 [36]	5	0	0	0	4	0	The Bilboquet device is simple, effective, and ensures stable fixation in complex PHFs, but it does not reduce the risk of AVN.
Doursounian et al., 2005 [37]	13	2	3	4	0	3	The Bilboquet device provides stable fixation in PHFs in the elderly, enables early mobilization, preserves bone stock, and restores humeral length.
Doursounian et al., 2000 [26]	5	1	2	3	0	2	Results compare favorably with other methods of internal fixation.
Doursounian et al., 2016 [27]	6	1	2	0	0	2	Ensures stable fixation in PHFs but does not prevent AVN. The cementless stem is easier to use but not superior to the cemented stem.
Bismuth et al., 2021 [28]	3	0	0	0	0	2	Both Bilboquet and locking plates yield good outcomes, but the Bilboquet device shows better mid-term functional results.
Doursounian et al., 1996 [25]	4	0	2	3	0	1	Provides stable fixation and enables an early mobilization.
Desai et al., 2021 [29]	2	2	1	0	0	2	The Bilboquet device offers a simple, stable fixation method with predictable tuberosity union and shoulder function, especially in patients under 70 years.
Doursounian et al., 2020 [38]	8	0	0	0	0	0	Excellent radiological and clinical evaluation.
Total *n* (%)	46 (19.6%)	6 (2.6%)	10 (4.3%)	10 (4.3%)	4 (1.7%)	12 (5.1%)	

## Data Availability

The data presented in this study are available upon request from the corresponding author.
